# Networking between community health programs: a team-work approach to improving health service provision

**DOI:** 10.1186/1472-6963-14-297

**Published:** 2014-07-09

**Authors:** Nathan J Grills, Robert Kumar, Maneesh Philip, Gillian Porter

**Affiliations:** 1 Redburn View, Landour Community Hospital, Mussoorie, Uttarakhand, India 248719; 2 Herbertpur Hospital, Vikas Nagar, Uttarakhand, India; 3 The Uttarakhand Cluster, OPEN Society, Old Rajpur Road, Rajpur, Uttarakhand, India; 4 The Nossal Institute of Global Health, 161 Barry St, Carlton, Victoria 3010, Australia

**Keywords:** Network, Partnership, India, Community health, Cluster, Health system

## Abstract

**Background:**

Networking between non-government organisations in the health sector is recognised as an effective method of improving service delivery. The Uttarakhand Cluster was established in 2008 as a collaboration of community health programs in rural north India with the aim of building capacity, increasing visibility and improving linkages with the government. This qualitative research, conducted between 2011-2012, examined the factors contributing to formation and sustainability of this clustering approach.

**Methods:**

Annual focus group discussions, indicator surveys and participant observation were used to document and observe the factors involved in the formation and sustainability of an NGO network in North India.

**Results:**

The analysis demonstrated that relationships were central to the formation and sustainability of the cluster. The elements of small group relationships: forming, storming, norming and performing emerged as a helpful way to describe the phases which have contributed to the functioning of this network with common values, strong leadership, resource sharing and visible progress encouraging the ongoing commitment of programs to the network goals.

**Conclusions:**

In conclusion, this case study demonstrates an example of a successful and effective network of community health programs. The development of relationships was seen to be to be an important part of promoting effective resource sharing, training opportunities, government networking and resource mobilisation and will be important for other health networks to consider.

## Background

Inter-organisation networking among non-government organisations (NGOs) is becoming increasingly recognised by donors and governments as an effective instrument to improve service delivery [1-5] (add in Kendall E). While published literature on the benefits of networking focuses on theory, there is little about the practice of networking at a community level in low- and middle-income countries (LMICs). A literature review by Kendall et al on the practise of networking uncovered seventeen published models of collaborative capacity building (Kendall et al. [5]). Another study from the USA outlines the importance of context - including history of collaboration, geography, community politics and values - for initiating effective collaborations. Both studies utilised case studies and articles exclusively from high income countries.

There is evidence from the literature that networking between local health organisations increases community awareness and participation [6], provides a forum for synthesising new evidence and ideas, amplifies messages for dissemination, improves efficiency and effectiveness of members through facilitation of learning, provides access to resources and gives an opportunity for members to improve inter-organisational linkages [2]. Some of the key factors that have been identified as contributing to the effectiveness of networks include: a clear mission and vision with realistic goals [1,6], effective leadership [6,7], shared decision making [7], built trust between members with successful negotiation of conflict [6] and an environment that promotes cohesion, task orientation and participation [7] while providing visible feedback on progress of group tasks [1].

The Uttarakhand Cluster (henceforth ‘the Cluster’) a program of the Community Health Global Network, is an example of one inter-organisational network. Established in 2008, it began as a collaboration of fourteen community health programs in rural north India which had the goal of working together to better respond to the significant health needs in the rural impoverished state of Uttarakhand. The main aims of this Cluster were to cooperate so as to build the capacity of community health programs through resource sharing and generation, increasing their visibility and more effectively linking with the government healthcare system. The Cluster has undertaken activities including collaborative research in tobacco control, health training on various areas, promoting disability inclusion and organising biannual knowledge transfer and exchange forums. This research aims to utilise the experience of the Uttarakhand Cluster as a case study to better understand the effectiveness of networking between community health programs. An initial review analysing the initial two years of the network has been published previously [8], while this paper expands on an understanding of the factors that contribute to the formation and sustainability of the Cluster.

## Methods

For this qualitative study, data was collected through Focus Group Discussions (FGDs) with key personnel from each of the Cluster member programs. Supplementary data was collected through a triangulation of indicator surveys undertaken with each member NGO, and participant observation which included the field notes of the Cluster facilitator and documents such as the regular reports sent to Cluster program members. Ethics approval was obtained from the La Trobe Ethics committee to undertake interviews, focus group discussions and participant observation and written informed consent was obtained from all participants.

The Uttarakhand Cluster holds biannual ‘Linking-2-Learn’ network workshops. Their primary function is to promote peer-to-peer education and collaboration. Each year, for a period of four years, two FGDs were undertaken in conjunction with these workshops. The first FGD was conducted in English with the eight members of the Uttarakhand Cluster board and the second was conducted in Hindi with the heads of member programs. Questions were standardised across discussions. The separation into two groups was intended to avoid a power imbalance and so promote open conversation. All member programs were invited to participate in the study to avoid selection bias. An outside research assistant was employed to limit observer bias. Each FGD was recorded, de-identified and transcribed by an outside independent researcher. There was significant demographical and status homogeneity between FGD participants within each group.

The analysis from 2008-2010 data was published previously [8], and here we analyse data from 2011-2012 inclusive. FGD data was coded and themes arising were analysed to explore what supports formation and sustainability of an effectively functioning network. The point of data saturation was indicated when there was little new data that arose from each additional survey and focus group. Observer data and indicator surveys were then considered to gain further insight into the activities of the Cluster and clarify any unclear FGD transcript findings. However, when undertaking the analysis, it became clear that data saturation was soon achieved in that the analysis of additional data was consistent with the previous themes. Additional surveys and FGDs would seemingly have added little novel information.

## Results

The overarching theme that emerged from the data was relationships. It was observed that the development of the Uttarakhand Cluster reflected Tuckman’s description of the stages seen in small group relationships: forming, storming, norming and performing (see Table 1) [9,10]. These four elements existing in the relationships within the Cluster assisted in describing the factors involved in the formation and sustainability of the Uttarakhand Network.

**Table 1:** Descriptions of stages in small group relationships

**Stage**	**Group structure**	**Task orientation**
Forming	Testing and dependence	Orientation to the task
	- Establishing interpersonal relationships	- Identification of task
	- Determining acceptable behaviours	- Establishing approach to task
	- Looking to leaders for guidance	- Identifying boundaries
	- Orientation	- Orientation
Storming	Intragroup conflict	Emotional response to task demands
	- Lack of unity	- Resistance to task demands
	- Tension and conflict	- Emotional response to discrepancy between task demands and personal needs
	- Control and competition between parties	
	- Sense of threat to independence and identity	
Norming	Development of group cohesion	Open exchange of relevant interpretations
	- Difficulties overcome	- Exchange of ideas
	- Developing new group norms	- Openness to other group members
	- Desire to maintain group	- Increased cooperation in working toward group tasks and goals
	- Cohesion and harmony	
Performing	Functional role-relatedness	- Emergence of solutions
	- Maturity	- Interpersonal structure becomes tool for task activities
	- Group focus on tasks	- Focus on collective performance
	- Adopt roles which promote group activities	- Effective and efficient functioning

(Adapted from [9]).

### Forming

The forming stage in small group relationships is a time of establishing interpersonal relationships and testing to identify boundaries [10]. Although this study focuses on the Cluster two years after its initiation, and factors involved in initial formation have been examined elsewhere [8], the ongoing growth of the network has resulted in further group formation. In 2008, at the formation of the cluster there were 14 programs who were signatories to Cluster formation document. Between 2008 and 2010 an additional 19 programs joined the cluster. In the two-year period of this study (2011, 2012) the Cluster grew from 33 to 40 programs. Participants viewed this as a great achievement:

‘A big point is that our project is a small project and we started this with passion and now we have grown with so many programs in the Cluster and for me this is a big achievement’ (F2011)

It was clear that the primary motivators that brought programs into the Cluster, and lead to the formation of the network, were relationships and a broadly similar faith understanding:

‘It seems to me a lot of people stay involved because people they know and respect, they know someone is involved… they trust those people they have a relationship with those people and they come’ (B2011)

‘If [we] didn’t have that faith maybe many of us wouldn’t have come’ (F2011)

Training opportunities and possible funding were noted to be a secondary factor in attracting new programs into the Cluster, and have resulted in sustainability of the cluster:

‘They have some hope you know that they will get help, either in the way of knowledge or training or maybe money. Because if they are small organisations, they always look forward to get something to continue their program… for that they come’ (B2011)

Despite this, location was one of the barriers to the ongoing growth of the network, with it being more difficult to get programs in the remote parts of the state involved. For example one FDG participant noted that although the Tehri Garhwal region is the biggest district of Uttarakhand, there were very few members from there because of the distance. This context limits the formation of the cluster more widely and the sustainability of the involvement of those programs who are in isolated areas.

The data demonstrated that the ongoing formation of relationships and new membership has played an important role in the network’s ongoing progress, with the possibility of further expansion driving the network forward.

### Storming

As with any group relationship, an element of storming emerged from the analysis of the data. Storming is a time of intra-group conflict or lack of unity where there may be disagreements and threats to independence or identity [10]. Storming, if not managed wisely, can threaten the sustainability of such a network. In the FDG data there was little direct conflict referred to, with only minor tensions or disagreements being voiced. There was some resistance to group decision-making and disunity concerning what the vision of the cluster was. For example, in regards to finances:

‘…Cluster is not a funding organization. So from the members there has to be some kind of fund, for which the board can decide in emergencies we can help’ … ‘This vision of Cluster is very specific that this is not a funding agency’ (F2012)

‘“One good thing is that there is no funding in Cluster” [response] “That is an obstacle” [response] “No it isn’t.”’ (F2011)

This reflected some division in opinions over the role of the Cluster in generating funding support. The facilitators also noted these ongoing tensions in their observations.

Transparency, or rather a perceived lack of transparency of processes, was another cause of some tension amongst focus group participants. This also reflects a concern about the lack of control that individual members have over the strategic direction of the network; a situation which is to be expected given the inevitable loss of program independence when a network is formed:

‘One of the things I feel is while selecting organisations there has to be more democratic process of doing that. Lots of time we see that some of the organisations are selected for something but we really don’t know how they came up. So it will be nice that more democratic process and transparent process shown that everyone is satisfied and this is why it was selected’ (F2012)

Although this analysis revealed extensive resource sharing (eg. training materials, program ideas and financial), there was also dissatisfaction over a perceived limited and unequal nature of that resource sharing. Some programs were sharing much and some not at all:

‘According to me that we don’t use each other’s resources. Only DVD has been used but the personal expertise haven’t been used’ (F2011)

‘Sometime we hesitate to share those skills and resources with others’. (F2012)

Also, despite reports suggesting increasing connections with the government, some members expressed frustration over a lack of interaction with the government, and this caused some disagreement over the direction that the cluster should take:

‘“I think we should involve more government people in our community programs and we should try to invite all the concerned department people” [response] “we tried to invite them but couldn’t do it” … “…many of the government officials wanted to be there” (B2011)

This area of advocating for more or less Government engagement naturally creates tension in relationships in the network because it threatens the identity and independence of an individual program.

Finally, tension was expressed around failing to facilitate inter-program training:

‘It was said that people go to each other’s organization and learn, that has also not been done. Maybe still we are hard and the impact will be that we will not be able to use each other’s expertise’ (F2011)

These factors were not presented as outright threats to or conflicts within the relationships of the group, but rather revealed some of the underlying disagreements over the strategic direction of the Cluster and barriers to progress. The cultural context in which the FDG was conducted meant conflict may have been hinted at and not spoken about directly. As noted in other networking research, successful negotiation of conflict is an important factor in network effectiveness [6]. Therefore, noting these areas of disagreement demonstrates a healthy stage and opportunity for the network relationships to develop and become sustainable.

### Norming

Over this study period, there was a particularly strong sense of the Cluster relationships coming together in a norming phase. In small group relationships norming occurs when difficulties are overcome and norms are developed to guide the groups standards, roles and cohesion [10]. The registration of the Uttarakhand Cluster as an official society in 2010 (Report 5^th^ L2L Dec 2010) was a defining feature of this norming phase.

A key part of norming is group cohesion. In the data, participants consistently noted that the Cluster had brought them together in a way they never had before:

‘Cluster has linked us together…literally taught us how to shake hands with each other…the Cluster has made us one’ … ‘Fellowship…has transformed our relations with others and we used to think that others are super and they are doing much more… but now we realise that we are one and we have one vision’. (F2011)

There was consistent evidence of increasing openness between programs with exchange of ideas and support:

‘Because of Cluster we are aware of what we do, who have a whole pool of consultants and experts in different areas and training also’. … ‘We know each other now and we can think to whom I can call this time’. (B2012)

‘That I think is one of the biggest things in the Cluster, we have a body to contact for a particular thing’. …‘Sometime we have some needs or problems and we don’t have anybody to go to. So through Cluster we understood whom to go to’. (F2012)

Also, a greater clarity about what brings them together and keeps them together was demonstrated with FGD participants mentioning their common values as facilitating open relationships. Thus common values were important not only in the formation, but alo in the sustainability of the cluster.

There was a sense of increasing purpose around the norms of Cluster operations expressed, particularly in the commitment to the primary tasks of the Cluster: Linking-2-Learn workshops and resource sharing between meetings.

‘Whether we are busy or don’t have time and somehow we take out the time … when we meet during the meetings or workshops… we come to know other Cluster members’. (B2012)

‘Through the Cluster we come to know that what resources are available and what new updates are, like in all mails sent’ … ‘Whenever I open my mails so I see that every time there are mails from [Cluster coordinator]’ (F2011)

The discussions also consistently demonstrated that Cluster members had developed a number of common purposes: mutual encouragement, mutual assistance and providing increased health coverage to needy areas.

‘I think we are able to encourage each other and that we are a part of one group and we can help each other, and smaller NGO can help bigger NGO and the bigger program can help smaller’ (B2012).

‘Now we have 38 programs we have more ability to be involved in and advice levels like that’. … ‘It means you are not alone when you face the government or it is not vulnerable as many programs work together a link with the State Health and Family Welfare and Cluster has a link with the government training program as well’ (B2012)

‘In the long term we should have strategies to cover all the districts of Uttarakhand because the districts of Uttarakhand are very remote and far’. … ‘To get whole Uttarakhand involved is something we still have as a challenge for us I think’ (B2012)

### Performing

Importantly, the Cluster relationships have reached a stage of maturity and are performing in relationship effectively and efficiently. The performance stage of small groups describes the stage when group energy is focused on the tasks determined by the group and the group’s interpersonal structure becomes the tool for task activities [10]. The FGDs demonstrated that the Cluster has been very active over the two-year study period. Here the motivating factors for joining the Cluster – resource sharing, training opportunities and networking were actualised. This was found to be important to attracting new members and for sustaining the involvement of existing members. FGD participants described examples of resource sharing and joint trainings:

‘Sharing through e-group those information are very vital very important and we are taking note of everything and finding good use’. (B2011)

‘Some graduate people wanted to go from our village… they wanted to do some training, so the CLHTC training was a good chance to send these girls and they started last year the course…so thanks to the Cluster’ (F2012)

‘We get opportunities for training as well. And we had never thought there will be a linking from Melbourne for training’ … ‘Since our staff got training in the Cluster on disability, since then we are quite serious on working in disability’ (F2011)

Various FGD participants also commented on the significant achievements made in networking, and the way the Cluster facilitated relationships with outside organisations such as donors and the government:

‘The disability program also we linked with the government. There were no programs linked to the government plans, and through the Cluster we reached the government. (F2012)

‘Yesterday I was discussing with our donor they came and they wanted to fund and they saw that [anti-tobacco DVD] and they were very happy because it’s very simple way to educate others. (B2012)

‘Usually it’s the government that tell what to do but what happened here was that the Cluster told the government that from where and what to start and policies they should have’ (F2011)

The data revealed that the cluster had developed a clearer leadership structure, which was required for the official registration with the government. It was also evident through the FGDs that the performance of the Cluster was attributed to the experience of members and effective leadership, which provided legitimacy to the group.

‘I think the effective leadership of… helps us to continue’ (F2012)

‘Everyone is having a background of more than 30 years in the committee. I like to know these things even government people like to know. When there is a problem people have got very good experience and background in these things’. (B2011)

This data demonstrates that the Cluster has been able to come together relationally and work effectively, with strengths in resource sharing, training, networking and leadership.

## Discussion

The results suggest that the Cluster networking model – whereby various programs come together into a collaborative network - has strong parallels with the formation and development of relationships within small group relationships. The elements of forming, storming, norming and performing were found to summarise the journey of this organisational network, ultimately arriving at place where the network was reported as performing. This provides a useful framework under which to understand the relational processes by which the Uttarakhand Cluster came into being, which was the objective of this paper. Key features of the Cluster, as encapsulated by this framework, included a common set of values that attracted people into the network (forming), provided a common identity for the network (norming), facilitated successful relationships and encouraged effective sharing of ideas and resources within the Cluster (performing). Although a number of other studies take a cross sectional exploration of factors contributing to successful networks, few studies examine the temporal changing nature of networks over time. Understanding this, which this study contributes to, is important for network formation and sustainability.

The importance of a common set of values and other contextual factors to promote relationships, is reported in studies from high income countries. Kelger et al. [11] describe how the community norms and values were important in the forming of a network and recruiting members. Similarly, Kendall et al. refer to stakeholder engagement, or development of key relationships, as key to effective collaborations. However, they stop short of defining the stages of such relationship building between stakeholders.

The sustainability formation and sustainability of the cluster depends on effectively managing these various stages of relationship formation. Once the storming had been worked through the norming and performing, which were evidenced in the two years under study, have clearly contributed to attracting additional members and maintaining the pre-existing membership. The importance of moving towards performing, and achieving successes, is supported by findings from other studies [2-4].

This research primarily utilised data from focus group discussions for analysis, two of the FGDs were conducted in Hindi, and an independent researcher did the translation and transcription, with a separate researcher performing analysis. During analysis there was some concern over the unknown quality of parts of the transcription, however given the nature of the discussion with multiple people interacting and the consistency of data across the FGDs, errors arising from the quality control were deemed minimal. Additionally triangulation with other data sources corroborated these findings.

The representative nature of the data could potentially have been limited by the fact that only two focus groups were undertaken each year, and that each focus group only had eight to twelve participants. However, when undertaking the analysis, it became clear that data saturation was soon achieved in that the analysis of additional data was consistent with the previous themes. Additional surveys and FGDs would seemingly have added little novel information.

While this research is specific to the context of the Uttarakhand Cluster and therefore is not automatically generalisable, as a case study the information gained from this analysis provides helpful insights into networking models. In particular, the applicability of small group dynamics to this organizational network may help other interested organisations improve their own processes as they set up networks and work towards sustainable networks. Furthermore, this study provides additional evidence that networks can ‘perform’ by effectively promoting cooperation with the government sector, generating additional resources for programs and promoting the learning of new ways to undertake community health work. These were the three elements of the Uttarakhand Cluster’s original aims. Such performance is important in sustaining the network and contributing to its growth. This research will also support the body of evidence around networks in health that is being accumulated, as further case studies are currently underway in Kenya, Bangladesh and Assam, India.

The demonstrated effectiveness of networking between community health programs is an encouraging result. A key finding from this case study, that could equally apply to other small NGOs, was the importance of relationships in the network to promote effective resource sharing, training opportunities, government networking and resource mobilisation. Models of public health network formation outlined by Kendall et al. [5] also find relationships as key to building effective collaborations in first world countries. This research may encourage small NGOs to consider how they can further improve their work through networking with other small NGOs in their geographical region. As in any small group relationship, networking requires commitment from organisations in terms of time and finances and development of a common goal or bond is an important relational characteristic that allows effective performance of the group. The nature of the relationship between small NGOs can be expected to shift between the different stages of group formation. However, if the cluster remains intact this case study demonstrates how the network eventually begins to perform. This research demonstrates that networks can have a very positive outcome for both the organisations involved and the intended beneficiaries and can facilitate opportunities that would not otherwise be available to small non-government organisations.

## Conclusions

This research demonstrates that networking between community health programs can be effective in promoting effective community health activity. The building of relationships between network members is key to facilitating the effectiveness of the network and in this case study we can see how the stages in small group relationships have played out to facilitate the achievement of the network goals of building capacity, increasing visibility and linking with outside organisations. Further research into the networking model in different countries will aid the ability to generalise the lessons learnt into other contexts.

## Competing interests

The authors declare that they have no competing interests.

## Authors’ contributions

NG conceived of the study, and participated in its design and coordination. NG oversaw the implementation of the study on the ground, assisted with the thematic analysis and preparation of the paper. RK helped plan the study, gain access to the network, arranged the fieldwork, undertake the research and review the data. MP helped plan the survey, facilitated the FGDs, arranged the fieldwork, compiled the data and reviewed the findings. GP reviewed the data, corresponded with the co-authors, lead the thematic analysis of the study and lead manuscript development. All authors read and approved the final manuscript.

## Pre-publication history

The pre-publication history for this paper can be accessed here:

http://www.biomedcentral.com/1472-6963/14/297/prepub
